# A Cross-Sectional Evaluation of Vitamin D Status and Ovarian Reserve Markers in Subfertile Women: A Single-Center Experience From Pakistan

**DOI:** 10.7759/cureus.21757

**Published:** 2022-01-31

**Authors:** Sibtain Ahmed, Ayra Siddiqui, Alinah Qureshi, Syed Sajjad Hussain, Imran Siddiqui, Uzma Imran

**Affiliations:** 1 Pathology and Laboratory Medicine, Aga Khan University, Karachi, PAK; 2 Medical College, Aga Khan University, Karachi, PAK; 3 Student Life Science Gateway, Mc Master University, Ontario, CAN; 4 Head Research and Development, Australian Concept Medical Center, Karachi, PAK; 5 Gynecology and Obstetrics, Australian Concept Medical Centre, Karachi, PAK

**Keywords:** anti-müllerian hormone, pakistan, subfertility, ovarian reserve, vitamin d

## Abstract

Objectives

This study was conceived with the objective of assessing the correlation between ovarian reserve markers and vitamin D deficiency (VDD) in a selected group of Pakistani subfertile women presenting at a specialized subfertility treatment centre. The measurements of antral follicle count (AFC), serum anti-Müllerian hormone (AMH), serum follicle-stimulating hormone (FSH), and serum vitamin D (VD) levels were the main tools used for the assessment of ovarian reserve.

Materials and methods

All female patients aged 18 to 45 years presenting with primary and/or secondary subfertility at the Australian Concept Medical Centre in Karachi, Pakistan from August 2016 to July 2021 were included in the study. The data of all eligible patients were recorded in the pre-defined Performa designed for this study. The Kruskal-Wallis test was applied to report the distribution of the data. The correlation between the categorical variables (25-hydroxyvitamin D [25-OHD] levels with AFC and AMH) was assessed using the chi-square test and Spearman correlation. The comparison was based on vitamin D levels grouped into three categories: deficiency (<20 ng/ml), insufficiency (21-29 ng/ml), and sufficiency (>30 ng/ml).

Results

One hundred ninety-nine cases were evaluated for AFC and hormone analysis. The mean age and BMI were 32.87±5.49 years and 28.27±4.97 kg/m^2^.VDD was noted in 127 (68.4%) cases. No significant difference was noted across BMI, age, duration of subfertility, AMH, and FSH across the VD categories. Moreover, a poor correlation was noted between VD, AMH and FSH on the scatter plot, between VD and FSH (r = −0.003, p = 0.966) and between VD and AMH (r = −0.068, p = 0.342), respectively.

Conclusions

This study showed a high frequency of VDD in Pakistani subfertile women, from a specialized subfertility center in the largest metropolis in the country. However, a statistically significant association was not found between the markers of ovarian reserve and VD, showing no ethnic differences in the native Pakistani population. Hence, VD supplementation is unlikely to have an impact on correcting the ovarian reserve status in subfertile women in Pakistan. However, this is a potential area of interest, and evaluation of other indices of reproduction/ovarian reserve and the effect of confounders is required to test this hypothesis longitudinally.

## Introduction

25-Hydroxyvitamin D (25-OHD) is a steroid hormone, rendering an upfront role in regulating the calcium and phosphorous levels in the body. Vitamin D (VD) exists in five different forms, i.e., D1, D2 (ergocalciferol), D3 (cholecalciferol), D4 (dihydroergocalciferol), and D5 (sitocalciferol). In humans, D2 and D3 are more prevalent [1]. Vitamin D deficiency (VDD) is highly prevalent in South Asia, especially in children and pregnant women [2]. The causes of this include limited exposure to sunlight, insufficient levels of VD in the diet, and a higher prevalence of naturally occurring dark skin, which requires a relatively long exposure time for VD synthesis [2,3].

VDD is common in women of childbearing age and is often linked to adverse maternal and neonatal outcomes [4]. According to Hogan et al., around 14 million pregnant women are subjected to acute maternal complications globally each year [5]. From a pathophysiological perspective, VD maintains the homeostasis of calcium and phosphate by regulating calcium absorption from the intestine and promoting skeletal utilization. Moreover, VDD is often associated with higher rates of small for gestational age (SGA) and low birth weight neonates and certain subsequent complications that can last into adulthood [6].

Subfertility is estimated to be prevalent at a rate of 12-14%, with VDD considered to be one of the causative factors [7]. Antral follicle count (AFC), anti-Mullerian hormone (AMH), and follicle-stimulating hormone (FSH) serve as established markers of ovarian reserves. However, AFC values can be subject to possible inter-observer or intra-observer variation inherent to sonographic measurements. Therefore, the results might be biased and inaccurately estimated.

On the other hand, AMH, a protein hormone secreted by granulosa cells of the ovaries which regulates early follicle development, is considered a better marker for ovarian reserve due to its standardization and convenience of testing [8]. It is expressed from the onset of puberty until menopause; however, it highly varies in each individual for unidentified reasons. To date, AMH has been found to be unaffected by the stage of the ovarian cycle, unlike FSH [9]. The AMH receptor-II gene promoter is linked with VD-triggered stimulation. In its active form, VD can upregulate AMH production. Hence, VD serves as a regulator for AMH concentrations in the blood [10].

Several studies [11,12] have advocated the impact of race and ethnicity on the evaluation of VDD and subfertility. Even though VDD is highly prevalent in Pakistan, there exists a substantial gap in the scientific literature regarding its significance in the female subfertile cohort. Longitudinal studies are required in subfertile women to elucidate the association between VDD and subfertility further. To address gaps in local data, this study was conducted to explore the association between VDD and ovarian reserve via measurements of AFC, AMH, FSH, and 25-OHD in Pakistani women.

## Materials and methods

A retrospective cross-sectional study was conducted at the Australian Concept Infertility Medical Centre after approval from the institutional ethical review committee (ACIMC-UI-07-2021). All female patients aged 18 to 45 years presenting with primary and/or secondary subfertility at the Australian Concept Medical Centre in Karachi, Pakistan from August 2016 to July 2021 were included in the study (n=301). Inclusion criteria were focused on subjects labelled as subfertile by the consultant gynecologist, based on the criteria of having failed to conceive after 12 months of no contraceptive use. Patients with missing data, i.e., BMI, duration of subfertility, age, AMH, 25-hydroxyvitamin D (25-OHD), AFC, and FSH were further excluded. Our study was registered with the NIH Clinical Trial Registry (Registration No: NCT05137964). This work has been reported in line with the STROCSS criteria [13].

The biochemical analysis was performed at the section of Chemical Pathology, Department of Pathology and Laboratory Medicine, Aga Khan University, Karachi. 25-OHD was analyzed by a chemiluminescence assay on the liaison XL (DiaSorin) analyzer. AMH was measured using an electro-chemiluminescence assay on the Roche Diagnostic e411 analyzer (Roche, Basel, Switzerland), while FSH quantification was done using the ADVIA Centaur FSH assay from Siemens Medical Solutions Diagnostics USA, Malvern, PA. Internal and external quality assurance were ensured according to the institutional protocol. Moreover, the laboratory is accredited by the College of American Pathologists (CAP) and the Joint Commission International (JCI), ensuring external quality assurance.

AFC was determined through a transvaginal 2D ultrasound of the pelvis. To reduce the bias, all ultrasound scans were conducted in the center using the same machine (Model 6v1: Sonoscape) and vaginal probe (3.5 Hz). Before the ultrasound procedure, patients were directed to have an empty bladder, and a standard ultrasound technique was used. Two values were obtained, one for each ovary, and an average was taken to obtain the final value.

The data of all eligible patients were recorded in the pre-defined Performa designed for this study. The Kruskal-Wallis test and one-way ANOVA were applied to report the distribution of the data. The correlation between the categorical variables (25-OHD levels with AFC and AMH) was assessed using the Chi-square test and Spearman correlation. The comparison was based on 25-OHD levels grouped into three categories: deficiency (<20 ng/ml), insufficiency (21-29 ng/ml), and sufficiency (>30 ng/ml) [14]. AMH was categorized as low (<1 ng/ml), low normal (1-2 ng/ml), normal (2-4 ng/ml), and high (>4 ng/ml). The third variable, AFC, was also classified into four categories: very low (<6), low normal (6-8), normal (8-10), high normal (10-12), and very high (>12). The p-value <0.05, was considered statistically significant. The statistical analysis was performed using the statistical software IBM SPSS Statistics for Windows version 23.0 (IBM Corp. Released 2015, Armonk, NY: IBM Corp).

## Results

A total of 199 cases fulfilled the predefined inclusion and exclusion criteria. VDD was noted in 127 (68.4%), insufficiency in 39 (19.6%), and sufficiency in 33 (16.6%) cases. The mean age and BMI of the group were 32.87±5.49 years and 28.27±4.97 kg/m^2^ respectively. No significant difference was noted across the BMI, age, duration of subfertility, AMH, and FSH across the 25-OHD categories as shown in Table 1.

**Table 1:** Comparison of demographic indicators and clinical characteristics with 25-OHD AMH: anti-Müllerian hormone, 25-OHD: 25-hydroxy vitamin D, FSH: follicle-stimulating hormone, BMI: body mass index

Variable	Total	25-OHD			p-value
		Deficiency (n=127)	Insufficiency (n=39)	Sufficiency (n=33)	
Age (years)	32.87±5.49	32.69±5.47	32.23±5.65	34.30±5.36	0.235
BMI (kg/m^2^)	28.27±4.97	28.54±4.99	28.54±5.29	26.87±4.38	0.213
Duration of subfertility (years)	7.75±4.93	8.06±5.11	6.74±4.18	7.76±5.01	0.349
AMH (ng/ml)	2.58±2.49	2.54±2.36	2.74±2.88	2.53±2.59	0.904^†^
FSH (mIU/ml)	8.28±4.59	8.24±4.15	9.49±6.37	7.03±3.32	0.075

On further stratification of AMH and AFC into fine grained categories, no significant association was revealed with the 25-OHD subgroup analysis as depicted in Table 2.

**Table 2:** Evaluation of AMH and AFC across the 25-OHD subgroups AMH: anti-Müllerian hormone, 25-OHD: 25-hydroxy vitamin D, FSH: follicle-stimulating hormone

Variable	Category	25-OHD			p-value
		Deficiency n(%)	Insufficiency n(%)	Sufficiency n(%)	
AMH (ng/ml)	Low	38(61.3)	13(21.0)	11(17.7)	0.780
	Low normal	21(65.6)	4(12.5)	7(21.9)	
	Normal	47(64.4)	17(23.3)	9(12.3)	
	High	21(65.6)	5(15.6)	6(18.8)	
AFC	Very low	21(72.4)	3(10.3)	5(17.2)	0.897
	Low normal	23(54.8)	10(23.8)	9(21.4)	
	Normal	50(65.8)	15(19.7)	11(14.5)	
	High normal	16(64.0)	5(20.0)	4(16.0)	
	Very high	17(63.0)	6(22.2)	4(14.8)	
Total		127(63.8)	39(19.6)	33(16.6)	

**Figure 1:**
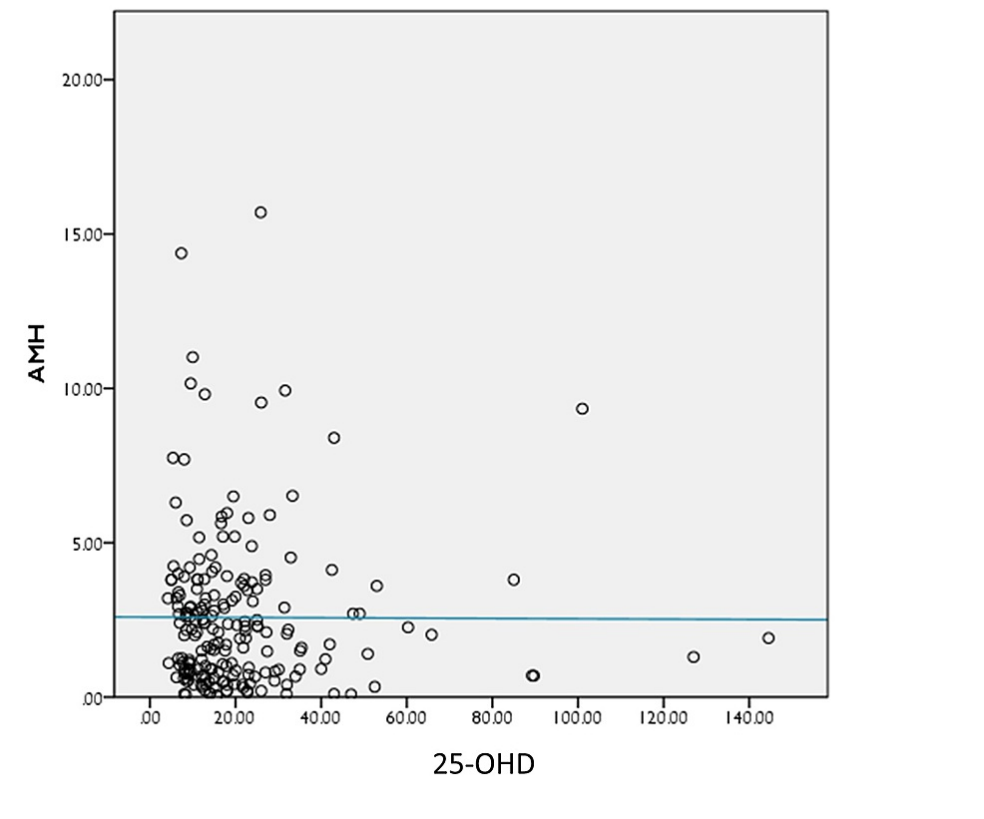
Spearman’s correlation between 25-OHD and AMH 25-OHD: 25-hydroxy vitamin D, AMH: anti-Müllerian hormone

**Figure 2:**
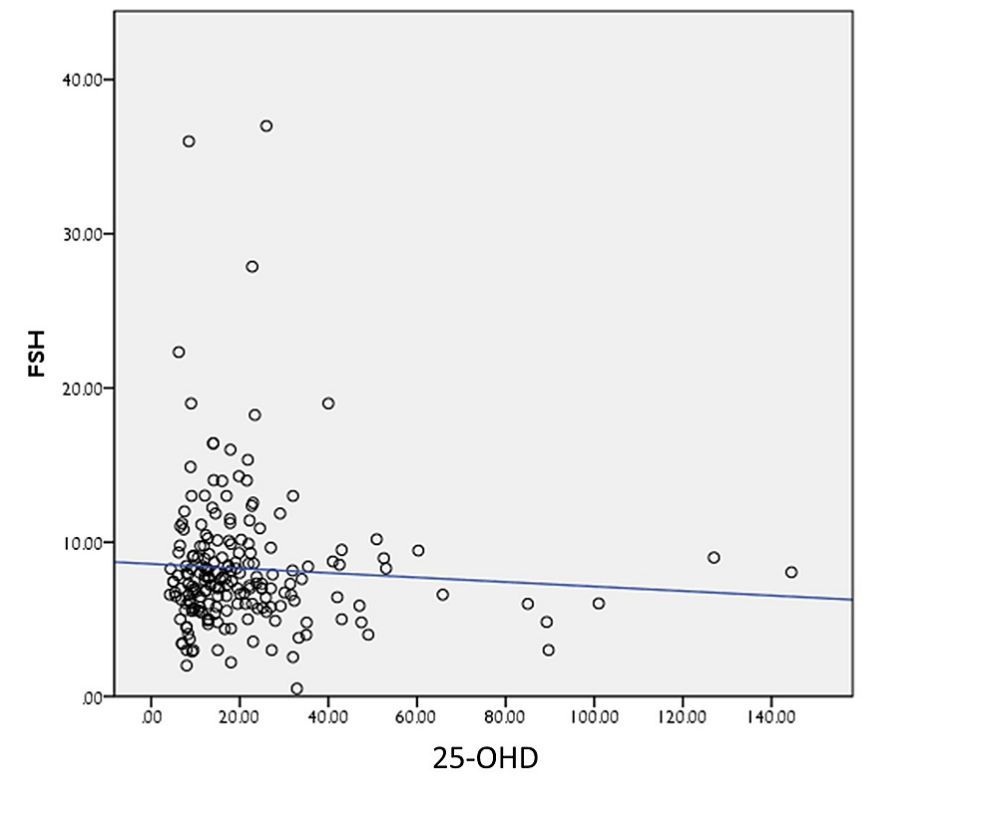
Spearman correlation between 25-OHD and FSH 25-OHD: 25-hydroxy vitamin D, FSH: follicle-stimulating hormone

Moreover, a poor correlation was noted between 25-OHD, AMH, and FSH on the scatter plot, between 25-OHD and FSH (r = −0.003, p = 0.966); and between 25-OHD and AMH (r = −0.068, p = 0.342), respectively, as shown in Figures 1-2.

## Discussion

Our results illustrate that the majority of the study participants were in the deficiency zone (68.4%). This, in accordance with published literature, demonstrates the high frequency of VDD in Pakistani women in the reproductive age group [15]. A study from Pakistan demonstrated that despite being a year-round sunny environment, VDD has been reported to be alarmingly high among women. A local study group has recorded that more than 90% of the pre-menopausal women had VDD with values lower than 20 ng/ml [16]. Similarly, another cross-sectional study from Karachi by Sheikh et al. showed a median concentration of VD as low as 18.8 ng/ml [17]. VDD in Pakistan has been associated with a lack of sun exposure, especially for women, due to religious obligations, poor dietary intake, and environmental factors, especially pollution [18].

With the growing frequency of subfertility in the metropolis, VDD as a potential contributor can be the missing link in the puzzle. However, a thorough literature review revealed substantial gaps in the literature. Therefore, this study was planned to collect baseline data from a specialized fertility treatment center and assess the relationship between VDD and indicators of ovarian reserve in subfertile women. The AMH levels undertaken in 199 cases correlated well with the AFC measurements.

The regression analysis of the study cohort revealed a poor correlation between subgroups of ovarian reserve biomarkers like AMH and/or AFC and different subgroups of VDD. Moreover, the evaluation of different ethnic groups in Pakistan in relation to VDD was also not able to show any significant correlation. A study by Alavi et al. evaluated 305 subfertile women, referred for in vitro fertilization, showed almost similar results, even after adjusting for baseline factors, authenticating our study findings and consistent with global literature for our part of the world [19]. At this point, regardless of fertility status, finding the VDD so prevalent in our cohort, the authors also felt, on prima facie, that there may be a need to relook and revise the normal reference limit for VD levels among Pakistani women.

The findings can postulate that VD supplementation is unlikely to improve AMH production. As proposed by various molecular characterization studies, VD receptor polymorphism did not lead to subfertility [20-21]. Hence, labelling VDD as a cause of subfertility is unjustifiable. There were certain limitations to this study. First, various environmental and biological factors contributing to subfertility were not considered. Furthermore, only the ovarian reserve markers were evaluated, while the status of other biochemical markers, like thyroid profile, serum prolactin or serum insulin, and complete hormonal status were not considered. Additionally, the impact of seasonal changes on VD levels was not accounted for. Moreover, the retrospective nature of the study and the small sample size may be factors limiting the power of the study. Hence, there is a likelihood that a significant correlation may arise with a larger sample size, emphasizing the need for prospective large-scale multi-center studies.

## Conclusions

This study showed a high frequency of VDD in Pakistani subfertile women, from a specialized subfertility center in the largest metropolis in the country. However, a statistically significant association was not found between the markers of ovarian reserve and VD, showing no ethnic differences in the native Pakistani population. Hence, VD supplementation is unlikely to have an impact on correcting the ovarian reserve status in subfertile women in Pakistan. However, this is a potential area of interest, and evaluation of other indices of reproduction/ovarian reserve and the effect of confounders is required to test this hypothesis longitudinally.
